# Cancer Across Domestic Animals: A Descriptive Review from the Veterinarian’s Perspective

**DOI:** 10.3390/vetsci13020167

**Published:** 2026-02-08

**Authors:** Antonio Giuliano, Rodrigo dos Santos Horta, Luca Santi Engel, Ayisa Rodrigues de Oliveira, Santiago Alonso, Celine Loubiere, Andrea Lombardo, Aldo Dal Prà, Felisbina Queiroga

**Affiliations:** 1 Harvest Veterinary Oncology Center (HVOC), Kwai Fung, Kwai Chung, Kowloon, Hong Kong SAR, China; 2 Department of Veterinary Medicine and Surgery, Veterinary School, Universidade Federal de Minas Gerais (UFMG), Belo Horizonte 31270-901, Brazilayisa.rodrigues@gmail.com (A.R.d.O.); 3 Department of Veterinary Clinical Sciences, Jockey Club College of Veterinary Medicine and Life Sciences, City University of Hong Kong, Kowloon, Hong Kong SAR, China; 4 Istituto Zooprofilattico Sperimentale del Lazio e della Toscana, M. Aleandri, Via Castelpuclci 43, 50018 Florence, Italy; andrea.lombardo@izslt.it; 5 Institute of BioEconomy-National Research Council (IBE-CNR), Via Giovanni Caproni 8, 50145 Florence, Italy; 6 CECAV, Department of Veterinary Sciences, School of Agricultural and Veterinary Sciences, University of Trás-os-Montes and Alto Douro (UTAD), 5001-801 Vila Real, Portugal

**Keywords:** cancer, domestic animals, neoplasia, genetic background

## Abstract

Cancer is a disease characterised by uncontrolled cell growth, that affects not only humans but also a wide range of animal species, and even plants. In this review, we explore and discuss the published literature about cancer across domestic animals. Most existing cancer across species studies have been conducted by researchers who are not familiar with the diagnosis and treatment of cancer in domestic animals, and the veterinary perspective is rarely included. Moreover, many scientists remain unaware of the value of clinical data obtained from spontaneous cancer in domestic animals. In this review, we examine, summarise, and comment on the available literature investigating cancer across different species, with a focus on domestic animals, including herbivores, carnivores, and omnivores. We also analyse the potential influence of body size, metabolism, environment, and genetic background on cancer risk across species. Understanding both cancer sensitivity and resistance mechanisms in different animals may help bridge current knowledge gaps between veterinarians, researchers, and human oncologists. Ultimately, these insights may support the development of more effective cancer prevention and treatment strategies in animals and humans, emphasising the One Health–One Cancer approach.

## Simple Summary

Cancer affects many domestic animal species, but its occurrence, tumour types, and clinical behaviour vary widely across them. Dogs and cats show high cancer incidence and share environmental risks with humans, making them valuable models for comparative oncology. Horses commonly develop skin tumours such as sarcoids, squamous cell carcinoma, and melanoma, while cancer appears less frequently in ruminants, partly due to shorter lifespans and underdiagnosis. Rabbits, rodents, reptiles, and birds also develop a diverse range of neoplasms, although available data remain limited and often fragmented. This review summarises the current knowledge on cancer across domestic animals, highlighting species-specific patterns, genetic and environmental influences, and their relevance for One Health and translational cancer research.

## 1. Introduction

Cancer is a complex disease influenced by multiple biological and environmental factors. In multicellular organisms, cell growth and cell-to-cell communication are tightly regulated to maintain tissue structure and function. When these mechanisms fail, abnormal cell populations can proliferate, invade surrounding tissues, and ultimately metastasize to distant organs, leading to life-threatening disease [1]. Somatic evolution may favour cancer cells, as they can outcompete normal cells in replication, survival, resource use, and other cellular activities [2,3,4].

Globally, cancer is one of the leading causes of death in humans, with more than 14 million new cases and over 8 million deaths annually [5,6]. Because cancer is strongly age-related, populations with increasing longevity show higher incidence rates, and it is anticipated that cancer will soon become the most common cause of death and a major global health challenge [5,6].

Cancer is not restricted to humans and companion animals; it has been reported across a broad range of species in the animal kingdom. Hemichordates appear to be the only group without documented neoplastic disease [5,7]. Even invertebrates can develop cancer, such as the well-characterised lymph gland tumours in Drosophila [8,9]. Tumours also occur in plants, although they are rarely lethal, as plant cells are confined within rigid cell walls that prevent invasion and metastasis [10].

Across species, cancer incidence varies widely [5,11]. Organisms throughout the Tree of Life have evolved diverse cancer-defence mechanisms, shaped by selective evolutionary pressures. In 1977, Sir Richard Peto proposed that cancer risk should scale with the total number of cells and lifespan; however, cancer incidence does not increase with body size or longevity across species, a phenomenon known as “Peto’s paradox” [5,7,12,13]. Although cancer becomes more frequent with advancing age, larger and longer-lived animals are not at higher risk than smaller ones [13,14]. Even within carnivores, large species such as bears, which live up to 40 years, do not show higher cancer rates than smaller carnivores like mustelids [15,16].

Research has therefore focused on understanding the factors that make some species more cancer-prone or cancer-resistant. A large survey of mortality data from 191 zoo species confirmed that animals with larger bodies or longer lifespans were not more likely to die of cancer [13]. Cagan et al. demonstrated that somatic mutation rates scale inversely with lifespan: cells of long-lived species accumulate mutations much more slowly than those of short-lived species [17]. Long-lived mammals have also evolved specific protective mechanisms such as reduced telomerase activity, restricted proliferation, and enhanced genome stability [14,18]. For example, elephants (Loxodonta Africana) acquired during evolution a high number of pseudogenes related to the tumour-suppressor gene protein 53 (TP53), a protein designed to protect the genome against replicative errors and dangerous mutations. This species has at least 20 copies of TP53, compared to a single copy in most mammals [1]. Similarly, long-lived bats such as Myotis that are particularly resistant to cancer, show additional copies of the tumour-suppressor gene F-box protein 31 (FBX031) together with down-regulation of certain genes that contribute to cancer resistance [18,19].

Although genetic predisposition is likely a major determinant of cancer risk across species, other factors, including diet, trophic level (herbivores, insectivores, carnivores, and carnivores consuming other carnivores), habitat/environment, and metabolic rate, likely contribute within an evolutionary multistage model of carcinogenesis [20,21]. Several studies suggest that cancer is more common in carnivores than in herbivores [13,20], with the highest rates reported in strict carnivores and in carnivores consuming other carnivores [13,20,22]. In humans, plant-based diets are associated with a reduced risk of certain cancers, particularly colon carcinoma [22,23]. However, extrapolating this concept to other species is complex. Non-human primates, despite predominantly plant-based diets, show variable cancer susceptibility, and certain species exhibit relatively high rates of colon cancer, illustrating that diet alone cannot explain species-level differences [5,24]. Being an herbivore could be associated with a lower accumulation of carcinogens from the environment compared to carnivores; however, different mechanisms to protect against cancer are likely to be adopted in different species, even if closely related. Most herbivores during evolution may have also inherited “a cancer protective genotype” in combination with a “vegan lifestyle” [13,20].

**Figure 1:**
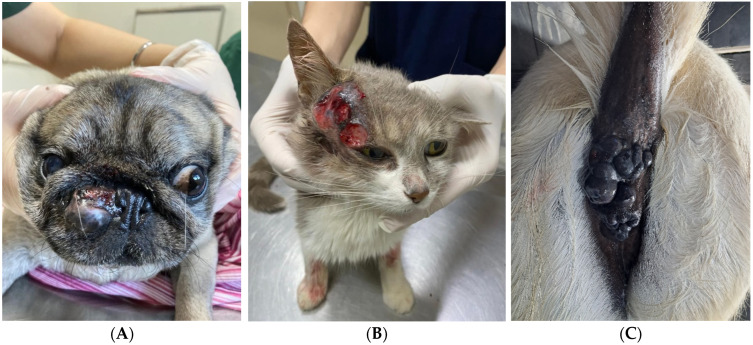
Cutaneous melanoma in the nasal planum of a dog (**A**), eyelid/preauricular alopecic region of a cat (**B**), and perineal region of a donkey (**C**).

Among domestic animals, cancer incidence is higher in dogs and cats, while herbivores such as cattle and horses show lower frequencies of specific tumour types (Table 1), with the biological behaviour of these diseases also differing across species (Figure 1) [13,23,25,26,27]. Cancer is one of the leading causes of death in both species, affecting approximately 1 in 3 dogs and 1 in 4 cats [28]. The development of structured veterinary cancer registries further strengthens opportunities for One Health-oriented comparative oncology [13,29]. Dogs and cats are often fed a diet with ingredients and nutrient composition that is quite close to human food. This could at least in part explain some similarities in the incidence of cancer. Another factor that should be considered is the environment as they often share the same urban and household environment as humans, making them similarly exposed to the same potential toxins and carcinogens [28]. Therefore, shared diets, lifestyles, and environmental exposures, particularly in urban settings, further reinforce the value of companion animals as natural models of environmental carcinogenesis.

When comparing cancer risk across species, mortality data alone may not reflect true incidence. Some cancers are less lethal or highly treatable, particularly in dogs and cats, where early diagnosis and therapy can result in a cure. In contrast, farm animals are rarely treated for cancer, making mortality data difficult to interpret. Studies relying solely on post-mortem findings may therefore underestimate incidence or misrepresent disease patterns if treatment history is not considered. Despite these limitations, the marked variation in cancer susceptibility across domestic animal species provides a valuable opportunity to investigate the natural mechanisms underlying cancer resistance and vulnerability. Elucidating these interspecies differences has the potential to inform both preventive and therapeutic strategies in veterinary as well as human oncology. In the following sections, we present a synthesis of current knowledge, tailored for a non-specialist audience, on cancer biology across domestic animals. For clarity, species are grouped according to veterinary convention into small companion animals (dogs and cats), horses, exotic species, and farm animals. Within each group, we highlight key similarities and differences that may guide future directions in comparative oncology research.

### 1.1. Cancer Patterns Across Domestic Animal Species

#### 1.1.1. Companion Small Animals: Dogs and Cats

Despite substantial gains in longevity in humans, dogs, and cats over recent decades, cancer remains a major cause of mortality in all three species [30,31]. Dogs are more likely than humans to die from cancer, and it is estimated that around 40–50% of dogs over ten years of age will develop cancer during their lifetime [32,33,34,35,36]. Although increased lifespan contributes substantially to cancer risk [34], many environmental and socioeconomic factors associated with oncogenesis in humans, such as diet, obesity, air pollution, and exposure to toxins, are similarly shared by companion animals [165,166,167].

Overall, the spectrum of neoplastic diseases differs markedly across the three species. In dogs, the most common tumours include sarcomas, mast cell tumours, mammary carcinoma, lymphoma, and oral melanoma, whereas in cats, lymphoma, squamous cell carcinoma (SCC), and mammary carcinoma predominate [37,38,39,61,168]. In contrast, carcinomas are the most frequent cancers in humans, particularly of the lungs, colon, prostate, and mammary gland. Apart from breast cancer, which is common to all three species, these carcinoma types are relatively uncommon in dogs and cats [38,39,40].

While carcinomas are the most common cancers in humans, dogs show a comparatively higher incidence of sarcomas such as subcutaneous soft tissue sarcoma, splenic haemangiosarcoma, and appendicular osteosarcoma [37,38,169]. As a rule, considering that mammalians have far more epithelial cells undergoing replication throughout life than mesenchymal cells, it would be expected that epithelial cells have more of a chance to accumulate mutations and thus give rise to more carcinomas than sarcomas. Because dogs and humans are exposed to similar environmental carcinogens, these differences in tumour distribution reinforce the possibility that dogs carry a stronger genetic predisposition to developing sarcomas in certain organs compared with people and other species [41]. In humans, sarcomas are rare and occur more frequently in children, further supporting a predominant role of inherited susceptibility; indeed, more than half of human sarcoma patients harbour germline variants in known cancer-predisposition genes [41,170].

##### Sarcoma

Canine osteosarcoma (OSA) represents one of the most robust spontaneous animal models of human high-grade osteosarcoma, sharing aggressive biological behaviour, early metastatic propensity, conserved histopathological features, and overlapping molecular alterations that support its translational relevance for therapeutic development [171,172].

Treatment of the most common appendicular osteosarcoma (OSA) in dogs is similar to that in people. Amputation combined with adjuvant chemotherapy using carboplatin remains the gold standard. Despite numerous studies investigating other adjuvant treatments, including different types of immunotherapies, survival outcomes have not improved over the past few decades. Prognosis remains poor, with only about 20% of dogs alive at 2 years [168].

Tumours of connective tissue—particularly soft tissue sarcomas—comprise a heterogeneous group in dogs and cats characterised by infiltrative growth patterns, variable metastatic risk, and complex tumour–stroma interactions, closely mirroring their human counterparts and offering valuable opportunities for comparative studies on local control and treatment response [173,174].

The importance of genetic risk is even more evident in dogs, as certain breeds display striking predispositions for specific sarcoma subtypes. For example, Labradors Retrievers are predisposed to splenic haemangiosarcoma, while Bernese Mountain Dogs and Flat-Coated Retrievers are particularly prone to histiocytic sarcoma, a tumour that is rare in the general canine population and even less common in humans [37,41]. Several studies have investigated the genetic basis underlying these breed-specific risks. For example, dogs carrying mutations in locus 11, particularly affecting CDKN2A and CDKN2B, have been associated with an increased likelihood of osteosarcoma and histiocytic sarcoma, while hemangiosarcoma has been linked to variants in TRPC6 and STX8 located on locus 5 [41,42,43,44,45].

Subcutaneous soft tissue sarcomas are relatively common in dogs and show no strong breed predilection, although large-breed dogs are somewhat over-represented. In contrast, these tumours are very rare in people. Despite their high incidence in dogs, they are infrequently a cause of mortality because most cases are effectively managed with complete surgical excision [172,173]. This remark highlights the potential bias when comparing different species’ post-mortem mortality data versus true prevalence in another living species population.

##### Carcinoma

Although sarcomas are over-represented in dogs, certain carcinomas are also clinically relevant and may serve as useful translational models. Mammary carcinoma is relatively frequent in both dogs and cats. As in humans, these tumours are hormone-dependent, and early ovariohysterectomy markedly reduces the risk, with incidence 7–10 times higher in non-neutered bitches and queens [39,46,175,176]. In certain countries or regions where neutering is not common, such as Brazil or parts of northern Europe, the incidence of mammary carcinoma is accordingly much higher [47,176]. Several features of dogs and cats’ mammary carcinoma resemble breast cancer in humans, including histological patterns, grading systems, and the influence of hormone receptors on tumour behaviour; however, as a general rule, feline tumours are often clinically more aggressive compared to canine tumours, and women are probably in between [48,176,177].

Transitional cell carcinoma (TCC) or urothelial carcinoma (UC) of the bladder and urethra are relatively common in dogs but rare in cats. In dogs, it occurs more frequently in certain small breeds, particularly the Scottish terrier [49]. In people, several predisposing factors, such as specific toxins and tobacco smoke, have been identified, but similar associations remain unclear in dogs [50]. As in humans, TCC in dogs typically shows an aggressive and invasive behaviour with a 50% of metastatic rate at necropsy [51].

Hepatocellular carcinoma (HCC) provides an example of divergent tumour behaviour across species. In humans, HCC is common and strongly associated with viral hepatitis and chronic alcohol consumption. In dogs, however, no viral or other clear aetiological factor has been identified [178]. While human HCC is highly aggressive, most canine HCCs are well-differentiated, slow-growing and associated with low metastatic potential. Surgical resection of massive or localised lesions is frequently curative, and long survival is common even in advanced cases [179].

Primary lung cancer illustrates another cross-species contrast. In humans, it is closely associated with environmental pollution, tobacco smoke, and radon exposure [180]. Although no direct causal link has been confirmed in dogs, environmental pollution is suspected to play a role [181]. Biological behaviour in both species depends largely on histological type and degree of differentiation. Treatment decisions follow similar principles: surgery is the main option for localised disease, whereas chemotherapy or target therapies have been used with controversial results in advanced cases in dogs [52,182].

Colon cancer, which is common in humans, is rare in dogs and cats [53]. While plant-based diets in humans reduce colon cancer risk, this factor does not explain the low incidence in carnivorous species such as dogs and cats [54].

##### Lymphoma and Lymphoproliferative Diseases

Lymphoma is an important neoplasm across species. Prevalence in humans and dogs is similar, at approximately 20–25 per 100,000 individuals, whereas cats exhibit a much higher prevalence (≈200/100,000) [55]. Interestingly, the most common type of lymphoma in dogs is multicentric diffuse large cell lymphoma, which closely resembles its human counterpart, whereas cats more frequently develop an intestinal form that predominantly affects the gastrointestinal tract and is relatively rare in humans [183]. Treatment strategies for high-grade lymphoma are broadly aligned across species and rely on multi-agent chemotherapy, such as the CHOP protocol, which includes cyclophosphamide, doxorubicin, vincristine, and prednisolone; however, veterinary protocols typically employ lower cumulative doses to minimise adverse effects and preserve quality of life [56]. In cats, the epidemiology and biology of lymphoma are strongly influenced by infectious agents, most notably feline leukaemia virus (FeLV). FeLV infection plays a well-established pathogenetic role in lymphomagenesis through viral integration, insertional mutagenesis, and chronic immune dysregulation, and has historically been associated with mediastinal and multicentric lymphoma in younger cats. Although widespread testing and vaccination have reduced the overall prevalence of FeLV-associated lymphoma, the virus remains a key determinant of lymphoma risk, anatomical distribution, and clinical behaviour in feline patients [184,185]. Leukemias are considerably less common than lymphomas in both dogs and cats. Interestingly, acute forms such as acute lymphoblastic leukaemia in dogs predominantly affect young patients, paralleling the pattern observed in humans. These cases are characterised by a rapid onset and highly aggressive clinical course [52].

##### Head and Neck Tumours

Oral tumours are also relevant in comparative oncology. In cats, oral squamous cell carcinoma (SCC) predominates and closely mirrors human disease in terms of biological behaviour. Dogs, however, show a high incidence of oral melanoma, a tumour that is rare in humans and cats [57,168]. Although anatomical pigmentation has been proposed as a contributing factor in certain dog breeds (e.g., Chow Chow and Dachshund), genetic predisposition is likely the main driver. In humans, the major risk factors for oral SCC include tobacco use, alcohol consumption, and papillomavirus infection, but such associations appear weak or absent in cats, which are not typically exposed to these carcinogens [58,168]. In addition to oral neoplasms, intranasal carcinomas represent an important epithelial tumour entity in dogs and occur with increased frequency in dolichocephalic breeds, a phenomenon that has been attributed to larger nasal cavity surface area and prolonged exposure of the respiratory epithelium to inhaled environmental carcinogens, further supporting the relevance of canine head and neck tumours as comparative oncology models [186,187].

##### Cutaneous Tumours

Cutaneous epithelial tumours are common in dogs and cats and are usually benign, much like follicular and basal cell tumours in humans [59]. In contrast, mast cell tumour (MCT) is the most common cutaneous malignancy in dogs and is less common in cats, presenting a more benign behaviour. Conversely, melanoma is one of the most frequent cutaneous malignancies in humans but is rare in dogs and very uncommon in cats [59]. Moreover, while melanoma in humans is strongly associated with UV exposure, this association is very unlikely in dogs, where cutaneous melanomas occur more commonly in dark-skinned, dark-haired animals, and usually display favourable behaviour with low metastatic rates [60,168].

##### Other Tumours

Tumours of the central and peripheral nervous systems in companion animals, including gliomas, meningiomas, and peripheral nerve sheath tumours, also display meaningful clinicopathological and histomolecular parallels to human disease; retrospective and immunohistochemical studies in dogs and cats have demonstrated comparable patterns of neural invasion, biological behaviour, and diagnostic challenges, reinforcing their relevance as comparative models for neuro-oncology [188,189,190]. Endocrine tumours, such as those arising from the thyroid, adrenal, and pancreatic glands, frequently present with hormonally mediated clinical syndromes and conserved signalling pathway dysregulation, yet remain inconsistently incorporated into comparative oncology frameworks despite their clear translational potential [191,192].

#### 1.1.2. Equines

Horses exhibit a markedly lower incidence of malignant neoplasia compared with dogs, cats, and humans. This phenomenon has been consistently documented in epidemiological studies and is often attributed to species-specific differences in lifespan, tumour biology, and overall cancer susceptibility [193,194,195,196,197,198]. In addition, horses develop a narrower spectrum of cancer types than dogs, cats, or humans. For this reason, the three most prevalent tumour types in horses will be discussed in greater detail.

The majority of reported neoplasms in horses originate from the skin or subcutaneous tissues, making cutaneous oncology a primary area of concern in this species. Unlike many other domestic animals, the overall risk of skin cancer in horses does not consistently increase with age. Instead, tumour distribution is strongly influenced by breed predispositions, genetic factors, viral infections, and environmental exposures [62,63,64,65,66,67]. Across studies, the three most frequently diagnosed tumours are sarcoids, squamous cell carcinomas (SCCs), and melanomas. Each of these exhibits distinct epidemiological patterns, biological behaviours, and clinical implications for affected horses [62,63,64,65,66,67] (Table 2).

##### Equine Sarcoids

Equine sarcoids are the most common skin neoplasm in horses and represent a significant clinical challenge due to their locally aggressive behaviour, despite the absence of metastatic potential [62]. They originate from the proliferation of dermal fibroblasts and are strongly associated with bovine papillomavirus (BPV), especially types 1 and 2, which are detected in a high proportion of lesions [68]. Although sarcoids can occur in any horse, several breeds, including Appaloosas, Arabians, and Quarter Horses, appear predisposed, supporting a genetic component to susceptibility [62,63,64,65,66,67].

Sarcoids are clinically heterogeneous and classified into six morphological forms: occult, verrucose, nodular, fibroblastic, mixed, and malignant/malevolent. Nodular, fibroblastic, and malignant/malevolent subtypes are generally considered more aggressive. Lesions frequently cause significant cosmetic and functional problems, especially when located around the head or periocular region, where they interfere with tack, field of vision, and overall welfare [69].

Histopathology remains the gold standard, though biopsies carry the risk of triggering lesion exacerbation or transformation [71,72]. PCR testing of superficial swabs for BPV DNA offers a reliable, non-invasive alternative, particularly for ulcerated lesions [73]. However, PCR sensitivity is lower in intact lesions, underscoring the need for more accurate diagnostic tools.

The concept of “benign neglect” is misleading, as untreated sarcoids can progress significantly. In one study of 42 untreated periocular sarcoids, 64% of horses were euthanised due to lesion severity [74]. Although some occult and verrucose sarcoids may regress spontaneously, one study reported regression in 65% of occult and 32% of verrucose sarcoids in Franches-Montagnes horses, though results may differ between breeds [75]. Additional research demonstrated a 14% spontaneous regression rate in a placebo group [76], further emphasising the need for more robust studies. While sarcoids of the head and neck are often considered more challenging to treat, a retrospective analysis found no significant association between sarcoid type or location and treatment outcome; however, horses with multiple lesions had lower success rates [77].

Treatment of sarcoids remains complex and often frustrating [74]. No single modality is universally effective, and treatment choices depend on lesion type, anatomical location, and accessibility [62]. Options include:

- Surgical Excision: Widely used but associated with high recurrence rates, particularly in periocular lesions [74]. Electrosurgery provides improved outcomes with success rates of 86.8% when wide margins are achieved [77]. Diode-laser excision yields approximately 83% success, although recurrences are more common at head and neck sites [78]. Cryosurgery, often applied after debulking, offers moderate success (70–80%) but is limited by frequent recurrences, especially in periocular sarcoids [74].
- Topical Treatments: Treatments like 5-FU and AW formulations have mixed results, with higher success rates when combined with other therapies [79]. Topical applications are often first-line for non-periocular lesions, but can cause collateral damage and require meticulous application to prevent spread and irritation.
- Radiotherapy: The most effective treatment for periocular sarcoids, though limited by cost and availability [80]. Low-dose brachytherapy has produced excellent outcomes, but access remains restricted.
- Other Treatments: Intra-lesional cisplatin, imiquimod, electrochemotherapy, and photodynamic therapy have demonstrated encouraging results, though further research is needed to confirm their efficacy [70,81,82]. Newer approaches like tigilanol tiglate and immunotherapies are emerging but are currently supported by limited data.

Early intervention is crucial for improving treatment outcomes, yet many sarcoids are left untreated until they progress, making management more difficult [62]. Given the complexity and variability of treatment responses, prompt diagnosis not only improves prognosis but also reduces long-term costs and complications associated with advanced disease.

##### Equine Squamous Cell Carcinoma (SCC)

Squamous cell carcinoma (SCC) is the second most common neoplasm in horses and typically affects poorly pigmented or depigmented skin, although it may arise in several other anatomical locations, including the eyes, genitalia, and stomach [83]. Chronic ultraviolet (UV) light exposure is a well-established risk factor, especially for tumours in sun-exposed areas with minimal pigmentation [84]. The eyelids, conjunctiva, and external genitalia are among the most frequently affected sites [85,86]. Genetic mutations, particularly in the TP53 gene, play a pivotal role in the pathogenesis of UV-induced SCC by impairing pathways responsible for cell-cycle regulation and genomic integrity [87,88].

Penile SCC, which accounts for 50% to 80% of external genital neoplasms, is strongly associated with chronic irritation and smegma accumulation, creating a pro-inflammatory environment favourable to carcinogenesis [86]. Viral involvement has also been implicated, as Equine papillomavirus-2 (EPV-2) is associated with both genital and gastric SCC. Upregulation of the EPV-2 E6 oncogene appears to disrupt host cell-cycle control, further contributing to tumour development [89].

Metastatic spread is an important feature of equine SCC, reported in approximately 6–18.6% of cases at the time of diagnosis [86,90]. Metastasis commonly involves regional lymph nodes; therefore, a full staging workup, including fine-needle aspiration of draining lymph nodes, is recommended to assess disease extent [91]. Wide surgical excision remains the mainstay of treatment, although recurrence is common, particularly in ocular and periocular lesions, where rates can reach up to 30.4% [92]. Adjunctive therapies like radiotherapy and chemotherapy are often employed to enhance long-term outcomes. Radiotherapy, especially strontium-90 plesiotherapy, achieves excellent success rates (83–100%) for periocular SCC [93]. Chemotherapeutic options include cisplatin beads and 5-fluorouracil, both of which have demonstrated effectiveness across multiple anatomical sites [81,94]. The overexpression of cyclooxygenase-2 (COX-2) in SCC has prompted interest in COX-2 inhibitors such as piroxicam and firocoxib, although further research is needed to clarify their therapeutic potential [95].

##### Equine Melanomas

Melanomas represent a major concern in equine oncology, particularly among grey horses, where prevalence is remarkably high. More than 80% of grey horses aged 15 years or older are expected to develop melanomas. Although more than 90% of equine melanomas initially present as melanocytomas (benign tumours), approximately 66% later progress to malignancy (melanoma) [96]. This progression is strongly linked to genetic mutations, most notably a 4.6 kb duplication within the STX17 gene, which contributes to the greying process and promotes melanocyte proliferation [97]. Additional mutations in the ASIP and MITF genes further increase susceptibility to melanocytic tumours in grey horses [98,99].

Unlike human melanoma but similar to the pattern observed in dogs, UV radiation does not appear to influence tumour development in grey horses. Despite having depigmented hair, grey horses retain dark epidermal pigmentation, which provides a protective barrier against UV exposure. In contrast, non-grey horses, such as bays and chestnuts, can also develop melanomas, but these tumours tend to have a more malignant behaviour and metastasize earlier than those observed in grey horses [100,101].

Equine melanomas are classified into four major categories: melanocytic nevus, dermal melanoma, dermal melanomatosis, and anaplastic malignant melanoma. In grey horses, multiple dermal melanomas or melanomatosis are common and may be locally invasive or metastasize to internal organs [102,103]. Diagnosis relies on a combination of fine needle aspiration, histopathology, and immunohistochemistry. Biomarkers, including RACK1, PNL2, and CD47, provide useful diagnostic support and may represent potential therapeutic targets in the future [96,104].

Treatment options include surgical excision, chemotherapy, and electroporation, along with emerging strategies such as immunotherapy and calcium electroporation. Notably, combining cisplatin with electroporation enhances intratumoural drug uptake and may be particularly valuable in anatomic regions where surgery is challenging and clean surgical margins are difficult to be acheaved [96,105].

#### 1.1.3. Ruminants

The specialised nature of modern ruminant farming, typically centred on a single production activity such as dairy or beef cattle, or rearing of sheep and goats, has made veterinary work highly sectorial, often focused on herd management, reproduction, and production efficiency [106]. Although veterinarians play a key role in disease detection, cancer diagnosis is rarely pursued in ruminants. This is largely because the economic value of individual animals seldom justifies the cost of advanced diagnostics, and because most ruminants in commercial systems have relatively short productive lifespans and are destined for slaughter. Consequently, tumours often go undetected, and comprehensive clinical or pathological data remain limited [107,108]. As a result, neoplasia in domestic ruminants is consistently reported as an accidental finding at slaughterhouses [27]; also, neoplasia seems to be less common than in companion animals, and the main types, along with their biological behaviour, are presented in Table 3 [108].

Mammary cancer in cows is notably rare (a finding recognised for nearly eight decades), and large anatomical surveys have shown that many suspected mammary lesions are inflammatory rather than neoplastic [109]. Nonetheless, ruminants can develop a variety of epithelial, mesenchymal, and mixed tumours, although the breadth of documented cases is still modest compared with other domestic species.

In small ruminants, tumours of the female reproductive tract are relatively frequent and are considered the second most common neoplasms after cutaneous tumours [107]. Broader pathological surveys reinforce that ruminants are susceptible to a wide spectrum of neoplasms. Another study analysing 59 suspected neoplastic samples from cattle and buffaloes reported that epithelial tumours accounted for 54.38%, mesenchymal tumours for 42.09%, and miscellaneous tumours for 3.5%; the overall malignancy rate was 43.86% [110]. Frequently identified tumours included papilloma, squamous cell carcinoma, fibroma, lymphosarcoma, adenoma, adenocarcinoma, melanoma, and myxoma, with several additional tumour types recorded sporadically [110]. Although cancer appears relatively infrequently in ruminants, partly due to biological factors and partly due to underdiagnosis, available evidence underscores the need for improved diagnostic reporting and more systematic surveillance across cattle, sheep, and goats. Since domestic ruminants are considered strictly as production animals worldwide, advanced and long-term oncologic therapies and clinical care have not been developed by veterinarians.

The goat represents the only domestic ruminant species occasionally raised as a companion animal, increasing its life span and clinical care needs. As already reviewed by Krus et al. in 2023, only a few papers discuss therapy and outcome of surgically treated domestic goats kept as companion animals [199]. Cutaneous and mammary carcinomas, together with tymomas, represent the most common types of neoplasia treated by surgical excision or cryotherapy; no data about non-invasive medical treatment are available. Also, in many countries, domestic ruminants are legally considered a food-producing animal species, regardless of their intended purpose (pet vs. production animal). The lack of data about legal, safe, and efficacious use of chemotherapeutics in ruminants still represents an issue of major concern in order to incorporate these drugs into clinical practice.

Therefore, any extralabel drug use in ruminants fails to satisfy legal and efficacy requirements, thus often resulting in unfeasibility in everyday veterinary practice.

##### Dairy and Beef Cattle

Dairy and beef cattle showed the highest incidence of neoplasms in the reviewed studies [116,117]. This is likely influenced by the very large global herd size, driven by increased production demands and the global rise in milk, dairy, and beef consumption. Among cattle, the organ systems most frequently affected by neoplasia are the integumentary system (particularly cutaneous papillomas) [27,108] and the alimentary system [27,118].

Virus-induced tumours represent an important subset of bovine neoplasms. Enzootic Bovine Leucosis (EBL) is a lymphoid malignancy caused by the Enzootic Bovine Leukaemia Virus (BLV), an exogenous retrovirus belonging to the genus Deltaretrovirus (family Retroviridae). This family also includes oncogenic viruses of significance in both veterinary and human medicine, such as human T-lymphotropic viruses 1 and 2 (HTLV-1, HTLV-2) and simian T-cell leukaemia viruses 1 and 2. BLV may be transmitted both vertically and horizontally; direct or indirect contact elicits viral spread through contaminated equipment or insect vectors. Although cattle are the primary host, experimental infections have also been reported in sheep and water buffaloes [119]. BLV infection leads to lymphoma (lymphosarcoma) after a prolonged, asymptomatic incubation period. Most infected animals remain clinically silent in an aleukaemic state; approximately one-third develop persistent lymphocytosis, and 5–10% progress to a severe, disseminated form of multiple lymphoid tumours [120]. Herds affected by EBL experience considerable economic losses due to increased mortality and morbidity, weight loss, and reduced milk yield [121]. As described before, no medical treatment is available for virus-induced neoplasia in cattle; EBL prevention is traditionally addressed with a population medicine approach, implementing specific state-instituted eradication programmes based on the “test and cull” strategy [120].

##### Small Ruminants

In goats, thymoma is among the most frequently reported neoplasms; however, unlike in other species, no consistent or distinct clinical syndrome has been clearly defined [200]. Lymphoma appears to be uncommon in this species. Tumours of the female reproductive tract can arise in the cervix, ovaries, uterus, vagina, and vulva, showing variable degrees of malignancy; approximately 10–50% of these are of smooth muscle origin. Cutaneous papillomas are the most common tumours affecting the skin and udder of goats. While outbreaks often affect multiple animals, no papillomavirus has yet been identified. Persistent papillomas on the udder may progress to squamous cell carcinoma [13,107,122,123].

Cutaneous melanocytic lesions are also reported in goats and sheep. In one small series of 15 goats with skin neoplasms, 40% were diagnosed with melanoma [124]. A larger 25-year retrospective study analysing 1146 goats submitted for biopsy or necropsy identified neoplastic lesions in 100 animals (8.7%) [125]. Despite the larger dataset, cutaneous melanomas were detected in only four goats (4%). Of these, three exhibited an epithelioid morphology (75%) and one a spindle-cell pattern (25%), with all lesions being pigmented. Lesions were located either at the coronary band (50%) or at the base of the horn (50%).

In small ruminants, cutaneous melanoma is considered highly malignant, locally invasive and metastatic, most commonly affecting lymph nodes (72%) and lungs (24%) [126]. Prognosis is usually fair to poor, particularly when regional or distant metastases are present. Surgical excision can be attempted, but the extent of tissue removal must be carefully evaluated, as local recurrence is frequent [124,126].

Sheep and goats may be affected by oncogenic retroviruses, most notably Jaagsiekte Sheep Retrovirus (JSRV) and Enzootic Nasal Tumour Virus (ENTV), which are associated with pulmonary and intranasal adenocarcinomas, respectively [127,128,129]. Both viruses are highly contagious and spread through direct contact and respiratory secretions. JSRV has also been detected in newborn animals, suggesting vertical transmission or exposure via infected colostrum [130]. Following an incubation period that may range from a few months to four years, infected animals develop progressive respiratory tumours: ENTV induces oncogenic transformation of the ethmoid turbinates mucosa, whereas JSRV transforms alveolar epithelial type II cells in the alveoli and Club cells (formerly named Clara cells) in the bronchioles, leading to progressive pulmonary adenocarcinoma. No medical treatment is available for JSRV and ENTV, and prognosis is often unfavourable [126].

#### 1.1.4. Pigs

A wide range of neoplasms has been documented in pigs and should be recognised as a potential cause of illness in individual animals. Excluding pigs raised as companion animals, pig farming cycles in the most common production systems involve rearing periods of less than one year [201]. Malignant cancers in young pigs are rare, although the true incidence may be higher due to underreporting [131]. Among the most common cancers in pigs are lymphoma, melanoma, nephroblastoma, and primary and secondary liver malignancies [131,132]. Congenital melanocytic neoplasia has also been described in the pig breeds Duroc, Iberico, and Nero Siciliano used in agricultural production systems. A higher incidence of this neoplastic disease can be assumed for small-scale farms with local breeds compared to professional pig husbandry due to potential inbreeding [133]. A case of abdominal squamous cell carcinoma in an adult domestic pig has recently been reported [202], but it is rarely reported in pigs. Comparative pathology recognises the value of porcine neoplasia as a model for human disease. Further insights into porcine cancers are needed to raise awareness, among practicing veterinarians, of the diagnostic processes and common outcomes when investigating neoplasia in pigs.

#### 1.1.5. Rabbits

The domestic rabbit (*Oryctolagus cuniculus*) is a lagomorph widely kept as a companion animal, with more than 50 different breeds derived from the European wild rabbit [134,135]. Epidemiological data remain limited, but neoplasia is mainly observed in middle-aged to older animals, with a mean age of onset between five and six years (Table 4) [136,137]. Because clinical signs may be subtle and owners frequently delay presentation, tumours in rabbits are often first detected as incidental findings during wellness examinations or work-up for non-specific signs [136,137].

##### Reproductive Tract and Mammary Neoplasia

Epithelial tumours of the reproductive tract are among the most frequently reported neoplasms in rabbits. Uterine adenocarcinoma is the predominant tumour in intact female domestic rabbits, particularly in animals older than three years, and should be considered a major differential diagnosis in does presenting with abnormal vulvar discharge, haematuria, reduced fertility, or vague systemic signs [135,136,138]. Abdominal ultrasonography is typically used as a first-line imaging tool to assess uterine enlargement and associated lesions, but definitive diagnosis relies on histopathology after surgical removal [136,137]. When disease is confined to the uterus, ovariohysterectomy is the treatment of choice and may be curative; however, prognosis becomes guarded when extra-uterine spread is present, highlighting the importance of early recognition and timely surgical intervention [136,137].

**Figure 2:**
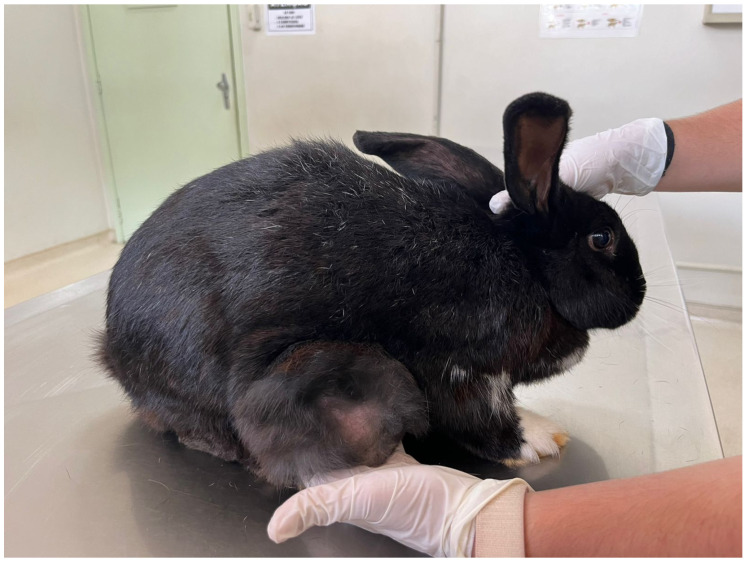
Mammary carcinoma in an intact female rabbit (*Oryctolagus cuniculus*), 5 years old. Macroscopic appearance of a large, firm mammary mass.

Mammary gland proliferative lesions, including hyperplasia and both benign and malignant tumours (Figure 2), are also overrepresented in females [136,137]. As in other species, complete surgical excision is preferred when feasible, and outcomes are influenced by tumour type, completeness of excision, and evidence of dissemination [136,137].

##### Cutaneous Tumours and Other Relevant Neoplasms

Trichoblastoma is considered one of the most frequent neoplasms in rabbits of both sexes [136]. Cutaneous masses are commonly amenable to complete excision, and prognosis is generally favourable when local control is achieved, although tumour type and anatomical location may impose surgical limitations [136,137]. In contrast, lymphoma is reported as the most common tumour in young rabbits, often presenting with non-specific clinical signs that may delay diagnosis [135,137].

Although less common, a variety of additional neoplasms have been documented, including thymoma [137,139], giant cell sarcoma [137], osteosarcoma [136], and odontogenic-like tumours [140], among others. In these less frequent entities, diagnosis is typically based on imaging to characterise the lesion and define surgical feasibility, followed by histopathology for definitive classification [136,137,140].

##### Conditions of Particular Clinical Relevance

Two specific conditions merit special mention due to their clinical implications. First, well-differentiated collagenous hamartomas (also termed fibromas or scleroderma-like lesions) may show a hormonal component. Regression following orchiectomy has been reported in males with elevated serum testosterone, although a true sex predisposition has not been conclusively demonstrated [136,141]. Second, myxomatosis, caused by Leporipoxvirus, is characterised by diffuse proliferation of undifferentiated mesenchymal cells with myxoid differentiation and carries an almost 100% mortality rate; it is also a notifiable disease in several countries [142]. Although not a true neoplasm, its tumour-like proliferative presentation is clinically relevant in the oncologic differential diagnosis of rabbits [142].

#### 1.1.6. Rodents

The group of domestic rodents is diverse and includes several species such as hamsters, mice, gerbils, rats, guinea pigs, and chinchillas [203]. These animals are widely kept as exotic pets due to their small size, ease of handling, and generally docile behaviour. A broad spectrum of neoplasms has been documented across rodent species, with many tumour types being age-related or strain-related, particularly in laboratory mice and rats (Table 5) [135].

##### Hamsters, Mice, Rats, and Gerbils

In hamsters, the skin is the most frequent site of neoplasia, with papillomas, squamous cell carcinomas, atypical fibromas, melanomas, and mast cell tumours commonly reported [143,144,145]. Mast cell tumours generally show a more benign behaviour in hamsters than in dogs. Cutaneous melanomas exhibit both breed and sex predisposition, occurring more often in male Syrian hamsters [144]. Atypical fibromas originate from ganglion-like cells within the dermis or subcutis and are observed predominantly in males over seven months of age. Lymphoma is another relevant neoplastic condition in hamsters and may present in multicentric, visceral, or epitheliotropic forms [143,144].

Across hamsters, mice, rats, and gerbils, several internal organs are also frequently affected by neoplasia, including the adrenal glands, ovaries, uterus, mammary glands, thyroid, pancreas, liver, and spleen [144,145]. Clinical signs vary widely depending on tumour type and biological behaviour.

In free-living rodents, mammary tumours are typically malignant and highly metastatic. In contrast, rats develop mammary tumours with very high frequency (up to 80% incidence), most of which are classified as mammary fibroadenomas. These tumours show marked hormonal influence and a decrease in prevalence after ovariectomy. A similar hormonal dependence is observed in rat pituitary adenomas, which are further promoted by obesity and high-calorie/high-protein diets. Rats may also develop neoplasms of the Zymbal gland (the sebaceous gland of the ear); although usually malignant, these tumours metastasise slowly [144].

Gerbils possess an androgen-dependent ventral abdominal scent gland that is prone to neoplastic transformation. These tumours may become malignant and metastasise, but castration is often curative due to hormonal influence. Testicular teratomas and ovarian granulosa cell tumours are also among the most frequently reported reproductive neoplasms in this species [144].

##### Guinea Pigs

Skin tumours are common in guinea pigs, particularly benign follicular neoplasms such as trichoepitheliomas and trichofolliculomas, as well as lipomas. Together, these lesions account for approximately 15% of all neoplasms in this species [144] and more than 60% of all externally palpable masses submitted for histopathology [146].

Mammary gland tumours are also frequent in guinea pigs, with about 50% showing malignant features. Most are adenocarcinomas, typically with a low metastatic rate and without sex predisposition (150:152).

Beyond cutaneous and mammary tumours, several other neoplasms are regularly reported in guinea pigs, including uterine leiomyomas, ovarian teratomas, thyroid carcinomas (both functional and non-functional), functional adrenal tumours, insulinomas, bronchogenic papillary adenomas, and lymphoma. Although less common individually, these neoplasms may have a substantial clinical impact, depending on anatomical location and functional behaviour [144].

##### Chinchillas

Chinchillas differ from other domestic rodent species in that neoplastic diseases appear to be rarely reported, raising the possibility of either true low predisposition or significant underdiagnosis in this species [144]. The available literature consists mainly of isolated case reports. Documented tumours include a vaginal leiomyoma and a uterine hemangioma in a clinically normal 12-year-old female [138]; a metastatic iridociliary adenocarcinoma in a 14-year-old male presenting with ocular rupture and pain [147]; and somatotroph pituitary adenomas in four females aged 4–18 years, all exhibiting neurological signs such as seizures, depression, ataxia, head pressing, and blindness [148]. A disseminated histiocytic sarcoma has also been described in a nine-year-old female with lethargy, anorexia, and dyspnoea, characterised by multiple skin nodules and widespread infiltration of neoplastic cells in the bone marrow, lung, heart, and spleen [149]. Although uncommon, these reports demonstrate that chinchillas are susceptible to a wide range of tumour types, underscoring the need for improved diagnostic awareness in this species.

#### 1.1.7. Reptiles

Reptiles have become increasingly prominent in the exotic-pet industry and now rank among the most commonly kept unconventional companion animals worldwide. Species such as pythons, bearded dragons, leopard geckos, corn snakes, crested geckos, boa constrictors, red-eared sliders, kingsnakes, chameleons, and green iguanas dominate this market [204]. Neoplastic diseases have been regularly documented across these taxa; however, their prevalence and biological behaviour vary considerably between reptile groups. While tumours are considered relatively uncommon in crocodilians and chelonians, they appear more frequently in lizards and snakes. Most neoplasms are spontaneous and age-related, although viral oncogenesis, genetic predisposition, and metabolic disorders may also contribute to tumour development in certain species [150,151].

The most commonly affected anatomical sites include the skin—where squamous cell carcinoma (SCC), papilloma, fibropapilloma, lipoma, and soft tissue sarcomas are frequently reported—followed by the kidneys (adenocarcinoma, adenoma, and nephroblastoma), oral and cloacal mucosae (SCC and polyps), liver (hepatoma, hepatocellular carcinoma and cholangiocarcinoma), and the hematopoietic and lymphoid systems (lymphoma and leukaemia) [150,151,152,153]. Beyond epithelial and mesenchymal tumours commonly observed in mammals, reptiles may also develop neoplasms that are rare or absent in other vertebrate groups. A notable example is chromatophoroma, an aggressive and highly metastatic tumour arising from pigment-producing chromatophores. Chromatophoromas have been documented in reptiles, amphibians, fish, crustaceans, and cephalopods, but are most frequently reported in snakes [154,155].

##### Lizards

**Figure 3:**
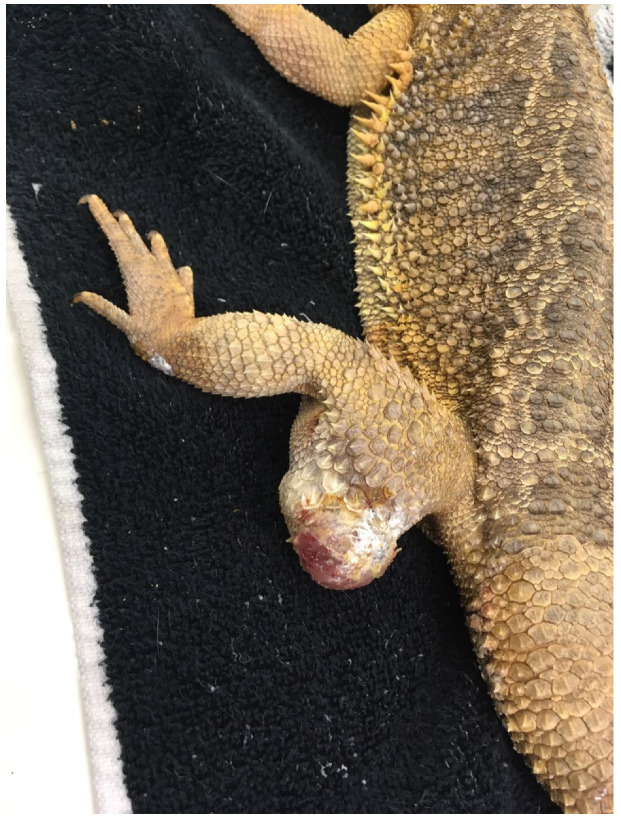
Soft tissue sarcoma in a bearded dragon (*Pogona vitticeps*). Macroscopic appearance of a large, firm, and infiltrative mass affecting the distal pelvic limb, with ulceration and disruption of the overlying scales. The lesion demonstrates the locally aggressive behaviour commonly associated with soft tissue sarcomas.

Lizards represent the reptile group most frequently described in oncologic case series. Squamous cell carcinoma is among the most common neoplasms, particularly affecting the oral cavity, skin, and cloacal region of species such as bearded dragons and iguanas [144,203]. Other tumours reported in this group include fibrosarcoma, osteosarcoma, and soft tissue sarcomas (Figure 3) [143,144]. Diagnosis typically involves physical examination combined with imaging modalities, such as radiography or computed tomography, to assess local invasion, followed by histopathology for definitive classification. Surgical excision remains the primary treatment option when anatomically feasible, with prognosis largely dependent on tumour type, location, and completeness of excision [143,144,203].

##### Snakes

In snakes, neoplasia appears to be less common overall than in lizards, but malignant tumours are proportionally more frequent [143,145]. Chromatophoromas, including melanophoromas, are characteristic of this group and may exhibit aggressive local behaviour and metastatic potential [154,155]. Other reported neoplasms include lymphomas and soft tissue sarcomas [143]. Clinical signs are often subtle or non-specific, contributing to delayed diagnosis. Imaging and histopathology are essential for tumour characterisation, while prognosis is frequently guarded due to late presentation and limited therapeutic options [143,145].

##### Chelonians and Other Reptiles

Neoplasia in chelonians (turtles and tortoises) is reported less frequently than in lizards and snakes, but a wide spectrum of tumour types has been described, including SCC, fibropapillomatosis-associated proliferative lesions, and visceral neoplasms (Table 6) [144,203]. Diagnostic and therapeutic approaches follow principles similar to those applied in other reptiles; however, shell involvement and anatomical constraints often limit surgical feasibility and adversely affect prognosis [144,203]. Reports of neoplasia in crocodilians remain rare and are largely restricted to isolated case descriptions [143].

##### Diagnostic, Therapeutic, and Prognostic Considerations

Diagnosis and treatment of neoplastic diseases in reptiles generally follow principles applied in dogs and cats; however, several reptile-specific factors must be considered. The low metabolic rate of reptiles increases sensitivity to medications, raising the risk of drug toxicity. As ectotherms, reptiles depend on appropriate environmental temperatures to maintain immune competence and ensure adequate therapeutic response. Additionally, the presence of a renal portal system necessitates caution during drug administration, as injections in the pelvic limbs may increase the risk of nephrotoxicity [156]. Consequently, chemotherapy and radiotherapy are rarely reported and lack standardised protocols in reptiles, making surgical excision the cornerstone of treatment whenever feasible. Overall prognosis is highly variable and depends on tumour type, anatomical location, stage at diagnosis, and the ability to achieve effective local control.

#### 1.1.8. Birds

A wide range of bird species is kept as pets, with psittacines (order Psittaciformes) being the most popular due to their sociable behaviour, intelligence, vibrant coloration, and vocal abilities [157]. As in other domestic species, neoplasia is regularly reported and is generally age-related, although viral oncogenesis and genetic predisposition may contribute to tumour development [157,158]. Overall, neoplasia prevalence has been estimated at 4.4%, with 2.3% of cases being malignant [159]. Rates are highest in Psittaciformes and lowest in Passeriformes [160]. In budgerigars, tumour prevalence varies widely, ranging from 17% [158] to as high as 66% in geriatric individuals.

The integumentary, gastrointestinal, and urogenital systems are among the most commonly affected. Clinical signs are often non-specific and may include anorexia, lethargy, coelomic distension, and respiratory compromise [158,160,161]. Testicular tumours are reported to be three times more common than ovarian or oviductal neoplasms, likely because males possess two functional gonads, whereas females have only one functional ovary.

**Figure 4:**
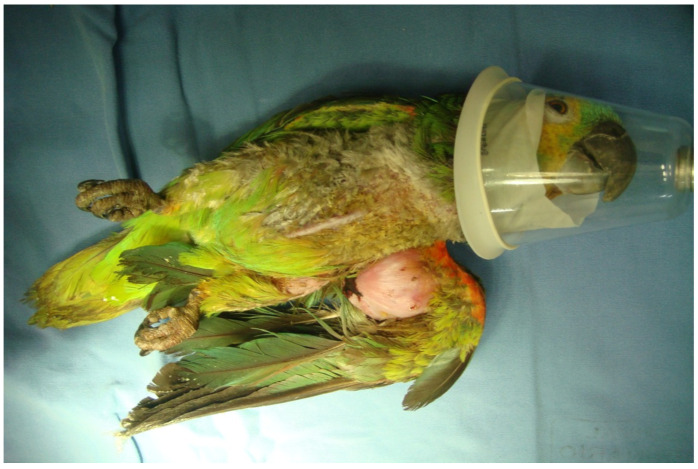
Squamous cell carcinoma in a blue-fronted Amazon parrot (*Amazona aestiva*). Macroscopic appearance of a large and infiltrative mass located in the humero-radio-ulnar joint, associated with marked feather loss and local tissue distortion. The lesion is consistent with the locally aggressive behaviour typically observed in avian squamous cell carcinoma.

Among the most frequently diagnosed tumour types, squamous cell carcinoma (SCC) (Figure 4) and papillomas are commonly found in the skin, oral cavity, oesophagus, crop, and cloaca, especially in psittacine species (Table 7). Proposed contributing factors include UV exposure, chemical irritants, genetics, diet, reused litter, infectious agents, hypovitaminosis A, and chronic wounds [158,162]. Lymphoma is also common in birds, particularly in Psittaciformes; it is apparently not linked to viral induction, as often happens in poultry with Marek’s disease [163]. Other tumours occasionally reported include adenomas and adenocarcinomas of the uropygial gland, air-sac adenocarcinomas, and feather folliculomas [158].

Treatment options are limited by challenges inherent to avian patients: small body size, difficult vascular access, high anaesthetic risk, high metabolic rate, and limited species-specific therapeutic data. When feasible, treatment follows the same principles applied in companion animals, with accurate diagnosis and staging being essential steps [160].

Two virus-induced neoplastic diseases remain of major economic relevance in poultry: Marek’s disease, caused by an oncogenic herpesvirus, and avian leukosis/reticuloendotheliosis, caused by retroviruses. These agents give rise to lymphoid and myeloid tumours, increased mortality, reduced productivity, and substantial economic losses for the poultry industry [164].

## 2. Conclusions

Cancer occurs across a wide range of domestic animal species, yet its incidence, tumour types, and biological behaviour differ substantially. These differences reflect complex interactions between genetic background, metabolism, life history traits, environmental exposures, infectious agents, and the degree of human intervention. Companion animals, particularly dogs and cats, exhibit high cancer incidence and share many environmental and lifestyle risk factors with humans, making them valuable comparative models for translational oncology. In contrast, ruminants and several exotic domestic species show lower reported cancer frequencies, largely influenced by short life expectancy, early slaughter, underdiagnosis, limited diagnostic work-up, and scarce post-mortem surveillance. Horses occupy an intermediate position, with distinct cutaneous neoplasms (sarcoids, SCC, and melanoma), representing important species-specific oncology models.

Across species, the current literature demonstrates large disparities in data availability. Companion animals benefit from robust clinical records and active veterinary oncology services, whereas production animals and exotic pets suffer from fragmented data and reporting biases. This lack of harmonised surveillance limits our understanding of true species-specific cancer risk and reduces opportunities to identify naturally occurring cancer-resistant phenotypes. Considering that domestic species live alongside humans, share diets and environments, and may even mirror human exposure to pollutants and carcinogens, a more integrated approach is essential to unlock their full value for One Health and comparative oncology.

From an evolutionary perspective, the wide variation in tumour susceptibility reveals that cancer resistance is not a binary trait but rather a spectrum shaped by selective pressures acting over millions of years. The existence of cancer-resistant phenotypes, such as low melanoma malignancy in grey horses or the near absence of mammary cancer in cows, has the potential to reveal protective mechanisms with relevance to human oncology. Conversely, species with high tumour burdens, such as dogs and cats, provide spontaneous models that closely resemble human disease in tumour heterogeneity, genetic drivers, metastatic patterns, and therapeutic response.

Strengthening the integration of veterinary and human oncology research will require coordinated efforts, including improved cancer registries across all domestic species, systematic necropsy and surveillance programmes in production animals, and broader inclusion of exotic species in comparative studies. Such initiatives will allow a clearer understanding of environmental, infectious, and genetic drivers of tumour development across species, supporting earlier diagnosis, better prevention strategies, and novel therapeutic approaches applicable to both veterinary and human medicine.

## Figures and Tables

**Table 1:** Comparative among species.

Species/References	Most Common Tumours	Key Particularities/Notes
Dogs [28,30,31,32,33,34,35,36,37,38,39,40,41,42,43,44,45,46,47,48,49,50,51,52,53,54,55,56,57,58,59,60]	Soft-tissue sarcomas; Hemangiosarcoma (spleen); Osteosarcoma; Mast cell tumour; Mammary carcinoma; Lymphoma; Oral melanoma	High overall cancer incidence (40–50% in dogs > 10 years); strong breed predispositions (e.g., Bernese: histiocytic sarcoma; Labrador: HSA); high incidence of sarcomas compared with humans; mammary carcinoma strongly hormone-dependent; share environmental carcinogens with humans.
Cats [37,38,39,55,57,58,61]	Lymphoma; Squamous cell carcinoma (oral/skin); Mammary carcinoma	Highest lymphoma incidence among domestic species; mammary carcinoma is very aggressive; oral SCC parallels human disease; fewer soft-tissue sarcomas compared with dogs; environmental tobacco exposure potentially linked to SCC.
Horses [62,63,64,65,66,67,68,69,70,71,72,73,74,75,76,77,78,79,80,81,82,83,84,85,86,87,88,89,90,91,92,93,94,95,96,97,98,99,100,101,102,103,104,105]	Sarcoids, Squamous cell carcinoma (SCC), Melanomas	Sarcoids = most common, BPV-related, locally aggressive; SCC linked to UV exposure and papillomavirus; melanomas extremely common in grey horses (80% > 15 years); tumour incidence NOT age-dependent; internal metastasis rare except in malignant melanomatosis.
Cattle [106,107,108,109,110,111,112,113,114,115,116,117,118,119,120,121]	Papillomas; SCC; Lymphoma (BLV-associated); Alimentary tumours	Neoplasia is relatively uncommon; BLV causes enzootic bovine leukosis with major economic impact; mammary carcinoma is extremely rare; most tumours are identified post-mortem; short lifespan reduces detection.
Goats [122,123,124,125,126,127,128,129,130]	Thymoma; Cutaneous papillomas; SCC (from chronic papillomas); Reproductive tract tumours; Melanoma (less common but aggressive)	Cutaneous papillomas are frequent, virus not identified; melanomas rare but malignant with lymphatic and pulmonary metastasis; reproductive tumours second most common; retroviral pulmonary/turbinate adenocarcinoma (JSRV/ENTV).
Sheep [127,128,129,130]	Reproductive tract tumours; Pulmonary adenocarcinoma (JSRV); Nasal adenocarcinoma (ENTV)	Oncogenic retroviruses play a major role; pulmonary adenomatosis is highly contagious; tumours are often detected late due to production systems.
Pigs [131,132,133]	Lymphoma, melanoma, nephroblastoma, liver cancer	Congenital melanocytic neoplasia described for the pig breeds Duroc, Ibérico, and Nero Siciliano; valuable comparative model for human disease
Rabbits [134,135,136,137,138,139,140,141,142]	Uterine adenocarcinoma (most common in adult females); Trichoblastoma; Mammary tumours; Lymphoma (young animals); Thymoma	Very high prevalence of uterine adenocarcinoma in intact females; mammary proliferative lesions are frequent; unique collagenous hamartoma (hormone-related); myxomatosis causes fatal mesenchymal proliferation (not a true tumour but clinically relevant).
Hamsters [143,144,145]	Skin tumours (papilloma, SCC, melanoma, atypical fibroma); Mast cell tumour; Lymphoma	MCTs are usually benign; atypical fibroma is more common in males > 7 months; cutaneous melanoma shows sex/breed predisposition; lymphoma in various forms.
Mice [135,144,145]	Mammary carcinoma; Lymphoma; Hepatic tumours	Strong strain-related tumour susceptibility; mammary tumours are often malignant with high metastasis; widely used as biomedical models.
Rats [144,145]	Mammary fibroadenoma; Pituitary tumours; Zymbal gland carcinoma	Very high incidence of mammary fibroadenomas (up to 80%); hormone-dependent and influenced by diet; Zymbal gland tumours are malignant but slow to metastasise.
Gerbils [144]	Ventral scent-gland tumours; Testicular teratoma; Ovarian granulosa cell tumours	Androgen-dependent gland neoplasia is common; responsive to castration; fewer systemic tumours vs. mice/rats.
Guinea pigs [144,146]	Trichoepithelioma; Trichofolliculoma; Lipoma; Mammary adenocarcinoma; Uterine leiomyoma	High proportion of benign skin tumours; mammary tumours 50% malignant but low metastasis; several endocrine tumours reported.
Chinchillas [138,147,148,149]	Leiomyoma; Hemangioma; Pituitary adenoma; Iridociliary adenocarcinoma; Histiocytic sarcoma	Very limited data—either genuinely rare or underdiagnosed; reported tumours involve the reproductive tract, ocular system, pituitary, and haematopoietic system.
Reptiles [150,151,152,153,154,155,156]	SCC; Papilloma; Chromatophoroma; Soft-tissue sarcoma; Renal tumours; Hepatic carcinoma; Lymphoma	High species-variation: rare in turtles/crocodilians, common in lizards/snakes; presence of chromatophoroma (unique to ectotherms); metabolic rate and renal portal system influence treatment.
Birds [157,158,159,160,161,162,163,164]	SCC; Papilloma; Lymphoma; Uropygial gland tumours; Air sac adenocarcinoma	Overall neoplasia prevalence ~4.4%; highest in Psittaciformes; testicular tumours > ovarian; viral tumours (Marek’s; Avian leukosis) important in poultry but not pet birds.

BLV = Bovine leukaemia virus; BPV = Bovine papillomavirus; ENTV = Enzootic nasal tumour virus; JSRV = Jaagsiekte sheep retrovirus; SCC = Squamous cell carcinoma; UV = Ultraviolet radiation.

**Table 2:** Equine tumours.

Tumour	Epidemiology/Risk Factors	Biology/Behaviour	Diagnosis	References
Sarcoid	Most common skin tumour in horses Strongly associated with BPV-1 and BPV-2 [68] Predisposition in Appaloosa, Arabian, Quarter Horse [62,63,64,65,66,67]	Locally aggressive, non-metastatic [62] Six clinical forms: occult, verrucosis, nodular, fibroblastic, mixed, malevolent Risk of worsening after manipulation/biopsy [71,72]	Clinical + morphological diagnosis Histopathology (gold standard) PCR for BPV (better in ulcerated lesions) [73] FNA with PCR shows promising potential	[62,63,64,65,66,67,68,69,71,72,73,74,75,76,77]
Squamous Cell Carcinoma (SCC)	Second most common tumour [83] UV-associated, especially in depigmented areas [84] Affects eyelids, conjunctiva, external genitalia [85,86] Viral involvement via EPV-2 [89]	Invasive local growth Metastases in 6–18.6% [86,90]—especially regional lymph nodes High rate of recurrence in the ocular region (up to 30.4%) [92]	Clinical examination and cytology Histopathology Regional lymph node FNA [91] Optional immunohistochemistry	[83,84,85,86,87,88,89,90,91,92,93,94,95]
Melanoma	Very common in grey horses: >80% ≥ 15 years [96] A total of 90% start benign (melanocytomas), but ~66% become malignant [96] Associated with STX17 mutation [97], ASIP and MITF [98,99] Not UV-related in grey horses [100,101]	Tumour of variable course They can become invasive or metastatic (lymph nodes, viscera) [102,103] Non-grey horses tend to develop more aggressive tumours	FNA, histopathology, IHC (RACK1, PNL2, CD47) [104] Clinical evaluation of the spread pattern	[96,97,98,99,100,101,102,103,104]

BPV = Bovine papillomavirus; BPV-1 = Bovine papillomavirus type 1; BPV-2 = Bovine papillomavirus type 2; EPV-2 = Equine papillomavirus type 2; UV = Ultraviolet radiation; FNA = Fine-needle aspiration; PCR = Polymerase chain reaction; IHC = Immunohistochemistry; ASIP = Agouti signalling protein; MITF = Microphthalmia-associated transcription factor; STX17 = Syntaxin 17; RACK1 = Receptor for activated C kinase 1; PNL2 = Melanocytic differentiation antigen PNL2; CD47 = Cluster of differentiation 47.

**Table 3:** Ruminants.

Species/Group	Most Common Tumour Types	Biological/Clinical Features	References
Cattle (Dairy and Beef)	Cutaneous papillomas Squamous cell carcinoma Alimentary tract neoplasms Lymphoma (BLV-associated) Fibroma, adenoma, adenocarcinoma, melanoma, myxoma (less common)	Highest reported incidence among ruminants, likely due to global herd size and intensive production. BLV-induced enzootic bovine leucosis is the most clinically significant neoplastic disease; long asymptomatic incubation; 1/3 develop persistent lymphocytosis; 5–10% develop disseminated lymphoma. Spread via direct/indirect contact or insect vectors. Causes substantial economic loss (mortality, decreased milk yield, comorbidities).	[27,108,110,116,117,118,119,120,121]
Small Ruminants–Goats	Thymoma (one of the most frequent) Cutaneous and udder papillomas (most common skin tumours) Reproductive tract tumours (10–50% smooth-muscle origin) Melanocytic tumours (rare but often malignant)	Papillomas may progress to SCC; no papillomavirus identified. Cutaneous melanoma: generally, highly malignant; common metastatic sites include lymph nodes (72%) and lungs (24%). Lesions may occur at the coronary band or horn base. Surgical excision is possible, but recurrences are common; prognosis is fair to poor when metastasis is present.	[13,107,122,123,124,125,126]
Small Ruminants–Sheep	Reproductive tract tumours (cervix, uterus, ovaries, vulva) Retrovirus-associated adenocarcinomas: Jaagsiekte Sheep Retrovirus (JSRV) → pulmonary adenocarcinoma–Enzootic Nasal Tumour Virus (ENTV) leads to intranasal adenocarcinoma	JSRV and ENTV are highly contagious; transmitted via close contact, respiratory secretions, and possibly colostrum (vertical transmission suspected). Incubation: months to 4 years. JSRV induces transformation of type II pneumocytes and Club cells leads to progressive pulmonary carcinoma. ENTV affects the ethmoid turbinate mucosa leads to nasal adenocarcinoma.	[127,128,129,130]

**Table 4:** Neoplasia in rabbits.

Species/Group	Most Common Tumours	Key Features	References
Rabbits	Trichoblastoma; Uterine adenocarcinoma; Mammary hyperplasia, adenoma, and carcinoma; Lymphoma (young animals); Thymoma; Osteosarcoma; Giant cell sarcoma; Odontogenic-like tumours	Neoplasia is most frequent in middle-aged to senior rabbits (5–6 years). Uterine adenocarcinoma is the most common tumour in intact females older than 3 years. Mammary lesions are common and may be benign or malignant. Lymphoma is most frequently reported in young rabbits. Collagenous hamartomas may regress after orchiectomy, suggesting hormonal influence. Myxomatosis causes a disseminated, undifferentiated mesenchymal proliferation with nearly 100% lethality.	[134,135,136,137,138,139,140,141,142]

**Table 5:** Neoplasia in rodents.

Species/Group	Most Common Tumours	Key Features/Particularities	References
Hamsters	Papilloma; Squamous cell carcinoma; Atypical fibroma; Melanoma; Mast cell tumour; Lymphoma	Skin is the predominant site of neoplasia. Mast cell tumours often behave more benignly than in dogs. Cutaneous melanoma shows male predisposition in Syrian hamsters. Atypical fibromas arise from ganglion-like cells and are more common in males older than 7 months. Lymphoma may present as multicentric, visceral, or epitheliotropic disease.	[143,144,145]
Mice	Mammary carcinoma; Pituitary tumours; Hepatic tumours; Lymphoma	Many tumours are age- or strain-related. Mammary carcinomas are highly malignant and metastatic. Pituitary tumours are influenced by hormonal status, diet, and obesity.	[135,144,145]
Rats	Mammary fibroadenoma; Pituitary tumours; Zymbal gland carcinoma; Thyroid tumours	Mammary fibroadenomas are hormone-dependent and may regress after ovariectomy. Pituitary neoplasia is associated with obesity and high-protein diets. Zymbal gland tumours are malignant but usually show slow metastatic progression.	[144,145]
Gerbils	Scent gland tumours; Testicular teratoma; Granulosa cell tumour	Ventral scent gland tumours are androgen-dependent and often respond to castration. Reproductive tumours are relatively common in this species.	[144]
Guinea pigs	Trichoepithelioma; Trichofolliculoma; Lipoma; Mammary adenocarcinoma; Uterine leiomyoma; Ovarian teratoma; Thyroid carcinoma; Adrenal tumours; Insulinoma; Lymphoma	Skin tumours account for approximately 15% of all neoplasms and over 60% of palpable masses. About 50% of mammary tumours are malignant, with no clear sex predisposition. Several endocrine tumours have been reported.	[144,146]
Chinchillas	Vaginal leiomyoma; Uterine hemangioma; Iridociliary adenocarcinoma; Pituitary adenoma; Disseminated histiocytic sarcoma	The literature is limited; neoplasia may be rare or underdiagnosed. Reported tumours usually affect older animals. Pituitary tumours are often associated with neurological signs such as seizures, ataxia, and blindness.	[138,147,148,149]

**Table 6:** Neoplasia in reptiles.

Species/Group	Most Common Tumours	Key Features/Particularities	References
Reptiles (Snakes, Lizards, Chelonians)	Squamous cell carcinoma; Papilloma; Fibropapilloma; Chromatophoroma; Soft tissue sarcoma; Hepatocellular carcinoma; Cholangiocarcinoma; Renal carcinoma, adenoma and nephroblastoma; Lymphoma; Leukaemia	Tumour prevalence is higher in lizards and snakes and lower in chelonians and crocodilians. Chromatophoromas are highly invasive and metastatic and occur most frequently in snakes. Tumour development may be influenced by age, viral oncogenesis, and metabolic disorders. Treatment is complicated by ectothermy, low metabolic rate, increased risk of drug toxicity, and the presence of a renal portal system.	[150,151,152,153,154,155,156,204]

**Table 7:** Neoplasia in birds.

Species/Group	Most Common Tumours	Key Features/Particularities	References
Birds (Psittaciformes, Passeriformes, and others)	Squamous cell carcinoma; Papilloma; Lymphoma; Uropygial adenoma and adenocarcinoma; Air sac adenocarcinoma; Folliculoma	Overall, neoplasia prevalence is approximately 4.4%, with 2.3% of tumours being malignant. Prevalence is highest in Psittaciformes and lowest in Passeriformes. Testicular tumours are three times more common than ovarian or oviductal neoplasms. SCC and papillomas frequently affect the oral cavity, crop, and cloaca. Lymphoma is common but usually not virus-induced, unlike Marek’s disease in poultry. Viral neoplastic diseases of major economic relevance include Marek’s disease and avian leukosis/reticuloendotheliosis.	[157,158,159,160,161,162,163,164]

## Data Availability

No new data were created or analysed in this study. Data sharing is not applicable to this article.
